# Results of native and transplant kidney biopsies of children in a single center over a 15 years period

**DOI:** 10.1080/0886022X.2017.1398094

**Published:** 2017-11-12

**Authors:** Emel Isiyel, Kibriya Fidan, Bahar Buyukkaragoz, Meltem Akcaboy, Yasar Kandur, Ipek Isik Gonul, Necla Buyan, Sevcan Bakkaloglu, Oguz Soylemezoglu

**Affiliations:** a Division of Pediatric Nephrology, Gazi University, Ankara, Turkey;; b Division of Pediatric Nephropathology, Gazi University, Ankara, Turkey

**Keywords:** Renal biopsy, native kidney, transplant kidney, histopathologic diagnosis, complication

## Abstract

Renal biopsy is an important diagnostic procedure in pediatric nephrology. This study retrospectively investigates the indications, results and complications in a single tertiary children’s hospital in Turkey. We evaluated the native and transplant kidney biopsies in Gazi University Pediatric Nephrology Department between 2001 and 2015. A total of 196 biopsies (144 natives and 52 transplants) were included into the study. The mean age of the patients was respectively 10.8 ± 3.5, 13.9 ± 1.5 years. The main indication for a biopsy was non-nephrotic proteinuria with or without hematuria (*n*= 35), followed by steroid-resistance nephrotic syndrome (SRNS) (*n* = 34) and Henoch-Schönlein purpura (HSP)–related proteinuria (*n* = 20) for native kidneys. We found that focal segmental glomerulosclerosis (FSGS) (11.7%) was the most common histopathologic diagnosis for native kidneys, but acute cellular rejection (7.6%) was the most common histopathologic diagnosis for transplant kidneys. The complication rate in our study was founded 6.6% totally. Different complication rates were found in other studies; however, we think that this difference comes from the inspecting methodology of the complications. Hence, we wanted to share our own experience in the context of other studies in the literature.

## Introduction

Renal biopsy can provide diagnostic precision, prognostic value and guide in therapeutic options for many renal diseases. In 1951, Inverson and Brun reported the first large series of needle biopsies of the kidney [1]. Although a renal biopsy is difficult in children because of variable sizes of kidneys and different levels of cooperation, especially with the possibility of ultrasound-guided biopsy enhanced with the latest technologies, clinicians can make a choice about biopsies undoubtedly [2]. The main indications for a renal biopsy in children include persistent microscopic hematuria with or without proteinuria, recurrent gross hematuria, steroid-dependent and steroid resistant nephrotic syndrome (NS), distinction of nephrotic syndrome/nephritic syndrome, persistently low C3 in acute post-streptococcal GN, acute renal failure of unknown etiology, cyclosporine protocol biopsy, renal involvement in systemic lupus erythematosus (SLE) and lastly, staging of HSP nephritis [3–5]. Although macroscopic hematuria has been described as the most common complication, the biopsy protocols and instruments used are not same in many publications to determine the complications and rates [2,6,7].

The purpose of our study was to analyze the indications, histopathological diagnosis and complications in patients who went through renal biopsy.

## Materials and methods

One hundred ninety-six native and transplant kidney biopsies were evaluated in Gazi University Pediatric Nephrology Department between the years 2001 and 2015. Although only one biopsy was applied to native kidneys, two or more biopsies were applied to the five transplant kidneys. Since the study was retrospective, informed consent or ethical committee approval was not inquired.

The indications for renal biopsy comprised steroid resistance or dependent nephrotic syndrome, persistent non-nephrotic proteinuria with or without hematuria, recurrent hematuria, HSP with proteinuria, systemic lupus erythematosus (SLE), acute renal failure (ARF) and chronic kidney disease for native kidneys. The indications for transplant kidneys were nephrotic proteinuria, persistent non-nephrotic proteinuria with or without hematuria, increased serum creatinine with or without abnormalities in dynamic scintigraphy and protocol biopsies.

The contraindications of kidney biopsies were small contracted kidneys, multiple bilateral cysts, uncorrectable bleeding diathesis, recent antiplatelet or anticoagulant therapy, severe thrombocytopenia, uncontrolled severe hypertension, hydronephrosis and horseshoe kidney.

All biopsies were performed by pediatric nephrologists with the aid of X-ray and real-time ultrasonography between the years 2001 and 2008. After 2008, the biopsies were achieved by both pediatric nephrologists and radiologists with the guidance of real-time ultrasonography.

Biopsies were applied in the supine position to the transplants and in the prone position to the native kidney patients. 16-gauge needles were used in biopsies of all transplants and natives that were ≤1-year old and 14-gauge needles were used in native biopsies >1-year-old patients. At least two cores were acquired from each patient.

Local anesthesia or sedation was used considering the age and compliance of patients.

All the kidney biopsies were studied under light microscopy, while immunofluorescence and electron microscopy were also performed when necessary.

After the biopsy, patients were instructed to remain supine at least 4-h. After 4 h, patients could only get up for using the toilet or for just eating. Vital signs were checked every 15 min for 2 h, every hour for 4 h, every 2 h for 6 h, and then every 4 h thereafter. All voided urine was inspected for macroscopic hematuria. Hemoglobin levels were checked routinely at approximately 4–6 and 10–12 h after the procedure.

An ultrasound was performed if the patient developed abdominal pain, gross hematuria, hypotension or a significant drop in hemoglobin. Each patient had an outpatient follow-up visit to a nephrologist in our center within 1–4 weeks after the renal biopsy.

## Results

A total of 196 biopsies, comprising 144 native and 52 transplants were included into the study. The mean age of the patients was respectively 10.8 ± 3.53, 13.9 ± 1.5 (range of 10 months until the age of 18) years for the native and transplant kidney. The distribution of biopsies by age is summarized in Table 1.

**Table 1.** The distribution of biopsies by age.

Age	Native	Transplant
<5	11	0
6–10	46	4
11–18	87	48

The most common indications are respectively non-nephrotic proteinuria with or without hematuria (35, 17.6%) and increased serum creatinine (32, 16.1%) in the native and transplant kidneys. The indications for a renal biopsy of native and transplant kidneys were summarized in Tables 2 and 3.

**Table 2.** Indications for a biopsy of native kidneys.

Indications	Frequency	Percentage (%)
Non-nephrotic proteinuria with or without hematuria	35	17.8
Steroid resistance NS	34	17.3
Steroid-sensitive NS	17	8.6
HSP	20	10.2
Isolated hematuria	16	8.1
Chronic kidney disease of unknown cause	9	4.5
SLE	7	3.5
Acute renal failure	5	2.5
Familial Mediterranean fever with proteinuria	1	0.5

**Table 3.** Indications for a biopsy of transplant kidneys.

Indications	Frequency	Percentage (%)
Increased serum creatinine	32	16.3
Increased serum creatinine and viremia of BK	3	1.5
Proteinuria	8	4
Protocol biopsies	7	3.5
Abnormalities in dynamic scintigraphy	2	1

After 2008, 120 (61.3%) biopsies were performed by both pediatric nephrologists and radiologists with the guidance of ultrasonography.

**Figure 1.**
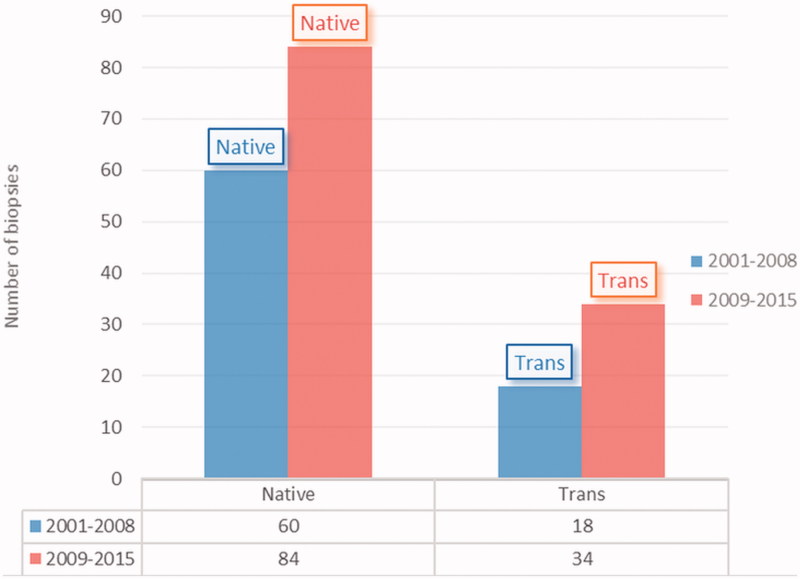
The distribution of biopsies by years.

The distribution of biopsies by years is shown in Figure 1. About 38.7% of biopsies were performed between 2001–2008 and 61.3% between 2009–2015.

Respectively FSGS (11.7%), HSP (10.2%), IgA nephropathy (9.1%) and acute cellular rejection (7.6%), acute T-cell-related rejection (4%), chronic allograft nephropathy (4%), and finally, acute tubular necrosis (4%) were the most common histopathologic diagnoses in the native and transplant kidneys. Histopathologic diagnoses of native and transplant kidneys were shown in Tables 4 and 5.

**Table 4.** Histopathologic diagnosis of native kidneys.

Diagnosis	Rate	Percentage (%)
Focal segmental glomerulosclerosis (FSGS)	23	11.7
HSP nephritis	20	10.2
Immunoglobulin A (IgA) nephropathy	18	9.1
Membranoproliferative glomerulonephritis (MPGN)	9	4.5
SLE	7	3.5
Minimal change disease (MCD)	6	3.5
Minimal mesangial proliferation, No feature on electron microscope/ Inadequate material to evaluate in an electron microscope	9	4.5
Alport syndrome	7	3.5
Chronic tubulointerstitial nephritis	8	4
Acute tubulointerstitial nephritis	6	3
Diffuse membranoproliferative glomerulonephritis	4	2
Hemolytic uremic syndrome	3	1.5
Acute postinfectious glomerulonephritis	2	1
Immunoglobulin M nephritis	2	1
Oxalosis	2	1
Nephronophthisis	2	1
Diffuse mesangial sclerosis	1	0.5
Lysosomal storage disease storage	1	0.5
Amyloidosis	1	0.5
Membranous GN	1	0.5
No definitive diagnosis	12	6.1

**Table 5.** Histopathologic diagnosis of transplant kidneys.

Diagnosis	Rate	Percentage (%)
Acute cellular rejection	15	7.6
Acute T-cell-related rejection	8	4
Chronic allograft nephropathy	8	4
Acute tubular necrosis	8	4
Toxicity of drugs	3	1.5
Ischemic injury in tubules	2	1
Tubulointerstitial changes	2	1
Acute antibody-related rejection	3	1.5
EBV related lymphoproliferative diseases	1	0.5
Thrombotic microangiopathy	1	0.5
No definitive diagnosis	1	0.5

Between 2001 and 2008, the FSGS rate in native kidney biopsy results was (*n*:8) 13%, compared to (*n*:15) 17.5% in 2009–2015. HSP nephritis and IgA nephropathy were (*n*:5) 8% and (*n*:4) 6% in 2001–2008; (*n*:15) 17% and (*n*:14) 16% in 2009–2015, respectively. Macroscopic hematuria, hematoma, AV fistula were seen as major complications in 13 patients (10 natives, 3 transplants) (6.6%). None of the patients with macroscopic hematuria and hematoma had transfusion required hemoglobin drop. In a patient with severe abdominal pain, an AV fistula was detected in the renal parenchyma and therefore, embolization was performed. There was no fatalities or loss of kidneys.

No significant difference was detected between complication rates in biopsies which were performed by a nephrologists or radiologist (*p* = .19)

The average number of the glomeruli was 21.7 ± 1.8. The average number of the core was 2.58 ± 0.6, while the minimum was 2 and the maximum was 4.

## Discussion

Renal biopsy is an established and effective diagnostic method in children and adolescents for many years [8]. The purpose of this study was to analyze the indications, histopathological diagnosis and complications of both native and transplant kidney biopsies in our center from 1 January 2001 to 31 December 2015.

The main indication for a biopsy was non-nephrotic proteinuria with or without hematuria (*n* = 35,17.8%), followed by the steroid-resistant NS (*n* =34, 17.3%) and HSP (*n* = 20, 10.2%) for native kidneys in our study. In one study, Bakr et al found that steroid-resistant nephrotic syndrome (28.4%) was the main indication [9]. While in another study, NS with hematuria and/or renal failure (32.1%) was presented as the main indication [10]. In addition to the aforementioned studies, Hussian F et al figured that the most common indication for a biopsy was steroid-resistant nephrotic syndrome and NS with atypical features [11]. Consequently, nephrotic or non-nephrotic proteinuria is the most common indication for biopsy.

At least a 10% rise in plasma creatinine from baseline, possible rejection or disease recrudescence was adopted as the criteria for biopsy of transplant kidneys by most centers [11]. In our study, increased serum creatinine (22.2%, *n* = 30) was the most common indication for transplant kidneys.

In our study, FSGS (*n* = 23, 11.7%) was detected as the most common histopathologic diagnosis for native kidneys. HSP nephritis, IgA nephropathies, SLE, MPGN, MCD, Alport Disease (total 35.3%) were observed as the other common diseases for native kidneys. In a study, Hussian et al, except MCD frequency, disclosed the similar results. In our study, acute cellular rejection was the most common histopathologic diagnosis for transplant kidneys.

After 2008, there was an increase in the number of biopsies compared to previous years in our study. Conveying of the patients to the pediatric nephrology department by the pediatricians in terms of signs and symptoms and facilitating biopsies with parallel to the technologic improvements may be the reason for this increase.

In our center, we administered intravenous sedation or verbal sedation with local anesthesia, so sedated patients are awake and dexterous shortly after the procedure. We did not perform any biopsy under general anesthesia. In different studies, it is noted that the majority of junior doctors, who are now trained in pediatric life, feel that short sedation is feasible and so the biopsies are easier to organize [12].

The complication rate was detected 6.6% totally. The number of complications reported in children following renal biopsy varied between 0–30% for major complications [13–15]. In some studies, macroscopic hematuria (4.5%) was the most frequent complication for native and transplant kidney. Sinha and colleagues described their complication rate of 12%. Most of the complications were macrohematuria (7%), (102 natives, 84 transplant kidneys) [15]. Rianthavorn et al founded major bleeding complications in three patients (1.3%) (277 native kidneys), two of them required blood transfusion and one underwent surgical repair of an injured renal artery. A higher incidence of macroscopic hematuria in children younger than 5 years and a higher incidence of perirenal hematoma in underweight children based on the weight for height Z-scores was reported in the same publication [2]. In a patient with severe abdominal pain, an AV fistula was detected in the renal parenchyma and embolization was performed in our study. Meager studies reported on AV fistulas in children and adolescents [16,17]. Standardization of devices and protocols are required to determine complication rates and incidence of kidney biopsies.

There was no significant difference in complication rates between biopsies performed by a nephrologists or radiologist (*p* = .19) in our study.

In conclusion, renal biopsy provides prognostic value and guide in therapeutic options for many renal diseases. Complication rates may be reduced by choosing appropriate needle and penetration depth according to the patient size and age.
